# Bayesian compositional generalized linear mixed models for disease prediction using microbiome data

**DOI:** 10.1186/s12859-025-06114-3

**Published:** 2025-04-05

**Authors:** Li Zhang, Xinyan Zhang, Justin M. Leach, A. K. M. F. Rahman, Carrie R. Howell, Nengjun Yi

**Affiliations:** 1 Biostatistics and Bioinformatics Facility, Fox Chase Cancer Center, Philadelphia, PA USA; 2 School of Data Science and Analytics, Kennesaw State University, Kennesaw, GA USA; 3 Department of Biostatistics, University of Alabama at Birmingham, Birmingham, AL USA; 4 Department of Medicine, Division of Preventive Medicine, University of Alabama at Birmingham, Birmingham, AL USA

**Keywords:** Bayesian models, Compositional data, Mixed model, MCMC, Microbiome

## Abstract

The primary goal of predictive modeling for compositional microbiome data is to better understand and predict disease susceptibility based on the relative abundance of microbial species. Current approaches in this area often assume a high-dimensional sparse setting, where only a small subset of microbiome features is considered relevant to the outcome. However, in real-world data, both large and small effects frequently coexist, and acknowledging the contribution of smaller effects can significantly enhance predictive performance. To address this challenge, we developed Bayesian Compositional Generalized Linear Mixed Models for Analyzing Microbiome Data (BCGLMM). BCGLMM is capable of identifying both moderate taxa effects and the cumulative impact of numerous minor taxa, which are often overlooked in conventional models. With a sparsity-inducing prior, the structured regularized horseshoe prior, BCGLMM effectively collaborates phylogenetically related moderate effects. The random effect term efficiently captures sample-related minor effects by incorporating sample similarities within its variance-covariance matrix. We fitted the proposed models using Markov Chain Monte Carlo (MCMC) algorithms with rstan. The performance of the proposed method was evaluated through extensive simulation studies, demonstrating its superiority with higher prediction accuracy compared to existing methods. We then applied the proposed method on American Gut Data to predict inflammatory bowel disease (IBD). To ensure reproducibility, the code and data used in this paper are available at https://github.com/Li-Zhang28/BCGLMM.

## Introduction

Compositional data exclusively depict relative abundances, such as, relative abundance of chemical elements in a mineral, the relative abundance of various nutrients in a food type, or the relative abundance of species in microbiome data [1]. In compositional data, the sum over the amounts of all components is fixed, so each component cannot vary independently [2]. This fixed-sum constraint makes modeling compositional data as predictors in generalized linear models inapplicable, as compositional data is not full-rank and the variance matrices are always singular. Thus, modeling compositional data and performing variable selection has been a barrier in recent years.

In 1982, Aitchison et al. laid the foundation for compositional data analysis by introducing a linear log-contrast model that employed the additive log-ratio transformation [3]. This transformation involves selecting one component as a reference and applying a log-ratio transformation to the remaining components, effectively addressing the constant-sum constraints. To address the singular challenge in a high-dimensional setting, where the dimensionality is comparable to or much larger than the sample size, various extensions have been proposed. Lin et al. proposed an $l_1$ regularization method for linear log-contrast model for variable selection [4]. They introduced a coordinate descent method of multipliers for efficient computation. The zero-sum constraint on the variable coefficients ensures that the model is equivalent to a log-contrast model and invariant to sample-specific scaling. Building upon these foundations, Zacharias et al. applied an elastic-net regularization to the logistic zero-sum model [5]; in 2019, Lu et al. extended their idea to generalized linear regression framework and developed a de-biased procedure to obtain asymptotically unbiased and normally distributed estimates [6]. Calle et al. performed variable selection through elastic-net penalization on generalized linear model containing all possible pairwise log-ratios [7].

In addition to the aforementioned techniques, more advanced and sophisticated methods have emerged to address the phylogenetic correlation among taxa in high-dimensional compositional microbiome data. For instance, Zhang et al. employed a Bayesian framework, utilizing a generalized transformation approach and a *z*-prior to effectively handle the constraints inherent to compositional data [8]. This approach further incorporates an Ising prior, designed to promote the joint selection of microbiome features that exhibit close genetic sequence similarity, providing a more comprehensive understanding of the microbiome structure. Furthermore, Zhang et al. have proposed a Bayesian approach that utilizes a structured regularized horseshoe prior for variable selection [9]. This approach also incorporates a soft sum-to-zero constraint to ensure compliance with the inherent compositionality of the data. By integrating the structured regularized horseshoe prior, this method facilitates effective variable selection while considering the potential dependencies and interactions among microbiome features.

All these methods are developed within the context of a high-dimensional sparse setting, where the underlying assumption that only a limited set of predictors influences the final outcome. However, in real-world scenarios, it is often more realistic to acknowledge that, alongside these large effects, there exists a multitude of smaller effects. Recognizing and incorporating these small effects can be instrumental in enhancing the predictive power of models.

To address this challenge, we propose the incorporation of generalized linear mixed models. In contrast to traditional sparse models, generalized linear mixed models effectively assume that each predictor contributes to the outcome, with effect sizes following a normal distribution. Drawing inspiration from the Bayesian Sparse Linear Mixed Model by Zhou et al. [10], which provides a hybrid approach combining the benefits of sparse and mixed models, we introduce the Bayesian Compositional Generalized Linear Mixed Models for Analyzing Microbiome Data (BCGLMM) to bridge this gap.

The BCGLMM consists of a standard generalized linear mixed model, with a random effect term and a structured regularized horseshoe prior applied to the compositional predictors. The structured regularized horseshoe prior can effectively capture the potential phylogenetic relatedness among taxa when selecting for moderate effects, while the random effect term can effectively accumulate the combined effects of the numerous small contributors for each sample. Further, to address the fixed-sum constraint in compositional data, we utilize a soft sum-to-zero restriction on coefficients through the use of prior distribution. This comprehensive model not only considers both major and minor contributors but also respects the unique compositional nature of the data, making it a valuable tool for predictive modeling in high-dimensional compositional microbiome research.

The paper is structured as follows. In Sect. Methods, we describe our proposed model, including formula specifications and prior distribution. We then present a performance evaluation of the proposed method on simulated data in Sect. Simulation studies and apply the method to identify bacterial genus associated with Inflammatory bowel disease (IBD) levels in American Gut Project questionnaire in Sect. Real date application. Section Discussion includes a discussion.

## Methods

### Model specification

Suppose ${\textbf {y}}=(y_1,y_2,\cdots ,y_n)$ is an *n*-vector response, and ${\textbf {X}}=(x_{ij})$ are $n\times m$ matrix of covariates with the constraints, $\sum _{j=1}^{m}x_{ij}=1$ and $x_{ij}\ge 0$. These covariates are referred to as compositional variables. In microbiome data, these covariates ${\textbf {X}}$ are relative abundances of *m* taxa, i.e., the observed counts divided by the total sequences.

The Bayesian Compositional Generalized Linear Mixed Models for Analyzing Microbiome Data (BCGLMM) is based on a standard generalized linear mixed model, which comprises three key components: the linear predictor $\eta$, link function *g* and data distribution *p* [11].

The linear predictor $\eta$ in this model comprises a linear function of the compositional variables and one random effect term:

1 $$\begin{aligned} \eta _i= & \beta _0+x_{i}\varvec{\beta }+u_i \nonumber \\ {\textbf {u}}\sim & MVN_n({\textbf {0}},{\textbf {K}}\nu ) \end{aligned}$$

where $\varvec{\beta }$ include the intercept $\beta _0$ and slope $\beta _1, \beta _2,\cdots ,\beta _m$. The term **u** represents subject-specific random effects that follows a distribution with mean 0 and variance **K**$\nu$. Following the principles of generalized linear mixed models, **u** are referred to as “random effects”, while $\varvec{\beta }$ are referred to as “fixed effects” [11]. The mean of the response variable is linked to the linear predictor through a link function *g*:

2 $$\begin{aligned} \mu _i = E(y_i|\eta _i)=g^{-1}(\eta _i) \end{aligned}$$

The distribution of the outcome data y depends on the linear predictor $\eta$ as well as a dispersion parameter $\phi$ (1 for Binomial and Poisson distribution), which can be expressed as:

3 $$\begin{aligned} Pr(y|\eta ,\phi )=\prod _{i=1}^{n}p(y_i|\eta _i,\phi ) \end{aligned}$$

Due to the constant-sum constraint inherent in compositional data, compositions cannot vary independently of each other. To accommodate the compositional nature of covariates **X**, a generalized transformation has been proposed [4, 6], and the linear predictor $\eta$ can be expressed as:

4 $$\begin{aligned} \varvec{\eta }=\beta _0+{\textbf {Z}}\varvec{\beta }^*+{\textbf {u}},\quad \sum _{j=1}^{m}\beta _j^*=0 \end{aligned}$$

with $\varvec{\beta }^*=(\beta _1^*,\beta _2^*,...,\beta _m^*)^T$ are the *m* regression coefficients and ${\textbf {Z}}=(z_1,z_2,\cdots ,z_m)=\left\{ log(x_{ij})\right\}$, are the $n\times m$ matrix of log-transformation of the original compositional data. In microbiome data, many observed counts of taxa are zero, which are typically replaced by a small pseudo-count, 0.5 or 0.5 times the minimum abundance before dividing by the sum to obtain relative abundance and log-transformation [6, 8].

Following Morris et al. [12], the sum-to-zero restriction $\sum _{j=1}^{m}\beta _j^*=0$ can be realized through “soft-centers” by assuming

5 $$\begin{aligned} \sum _{j=1}^{m}\beta _j^*\sim N(0,0.001*m) \end{aligned}$$

which tightly constrains the sum of $\beta ^*$ to be within some epsilon of zero.

### Prior distribution

A critical issue arises in microbiome data where the number of taxa, *m*, is typically comparable to or larger than the limited sample size, *n*. Conventional methods can be nonidentifiable when estimating the parameters. To address this, a Bayesian approach can be used by specifying prior distributions on the parameters [11].

For the intercept and the dispersion parameter, relatively flat priors can be utilized. For instance, in the context of scaled data, assumptions such as β_0_~*t*(3,0,10) and ϕ_0_~half-*t*(3,0,10). In case where the model involves other covariates, we can use weakly informative priors, for example Cauchy (0, 2.5) [13]. For the compositional coefficients, we use the regularized horseshoe prior, a recently developed sparsity inducing prior for high-dimensional models. The regularized horseshoe prior can be expressed as [14]:

6 $$\begin{aligned} & \beta _j^*|\lambda _j,\tau ,c\sim N(0,\tau ^2\tilde{\lambda }_j^2) \nonumber \\ & \quad \tilde{\lambda }_j^2 = \frac{c^2\lambda _j^2}{c^2+\tau ^2\lambda _j^2}\nonumber \\ & \quad \lambda _j \sim \text {half-Cauchy}(0,1) \nonumber \\ & \quad \tau \sim \text {half-Cauchy}(0,1) \nonumber \\ & \quad c^2 \sim \text {Inv-Gamma}(\nu /2,\nu s^2/2) \end{aligned}$$

There are three types of parameters in regularized horseshoe prior: the global shrinkage parameter $\tau$, the local shrinkage parameters $\lambda _j$, and the slab parameter $c^2$. The global shrinkage parameter $\tau$ shrinks all the coefficients $\beta _j^*$ toward zero, while the heavy-tailed half Cauchy priors for the local shrinkage parameters $\lambda _j$ allow some of coefficients to escape the shrinkage. The slab parameter $c^2$ serves to provide some shrinkage for large coefficients, ensuring that the model is always identifiable. The above regularized horseshoe prior ensures that small coefficients are heavily shrunk towards zero while large coefficients remain large.

Following Piironen and Vehtari [14], we set $\nu =4$ and $s^2=2$ for the slab parameter $c^2$, resulting in a weakly informative prior on $c^2$. In previous work [9, 15], we have shown that the global shrinkage parameter $\tau$ with a heavy-tailed Cauchy prior is minimally impacted by the choice of the scale; in this article, we use the default setting with a scale of 1.

### IAR model for phylogenetic relatedness

As discussed in the regularized horseshoe prior, we understand that it is the local scale $\lambda _j$ that determines the shrinkage severity for the $\beta _j^*$ estimates. Phylogenetic related taxa should have similar effects, and these similarities can be modeled by dependence upon the prior distribution of local scale $\lambda _j$ so that species with similar genetic sequence will have similar chance of being selected [9, 15].

To model these spatially structured priors, Zhang et.al [9, 15] proposed employing the intrinsic autoregressive (IAR) model, a special case of the CAR model [16, 17]. Despite having an improper distribution, the IAR model enables the modeling of stronger dependencies among variables compared to traditional CAR models and has been effectively used as a prior distribution [17, 18].

To work with these models, $\lambda _j$ are log-transformed, with $\psi _j$=log($\lambda _j$), and $\psi _j$ $\in$ $(-\infty ,\infty )$. $\psi$ thus follows a normal distribution, and with the IAR model, each $\psi _j$ varies about the mean of its correlated neighbors rather than a global mean [9, 15]. The pairwise difference formula can be expressed as:

7 $$\begin{aligned} p(\psi )\propto exp\left\{ -\frac{1}{2}\sum _{i\sim j} w_{ij}(\psi _i-\psi _j)^2\right\} \end{aligned}$$

Here, $i\sim j$ indicates taxa *i* and *j* i.e., with their weight $w_{ij}$. ${\textbf {W}}=(w_{ij})$ is the weight matrix, measuring the relatedness among taxa. The values of this matrix are determined through the analysis of a similarity matrix, assigning greater weights to taxa exhibiting significant phylogenetic similarity compared to those with relatively lower similarity. Specifically, we can construct the similarity matrix via the following transformation of taxonomic distance metrics:

8 $$\begin{aligned} {\textbf {W}}=-\frac{1}{2}({\textbf {I}}-\frac{1}{m} {\textbf {11}}')D^{(2)}({\textbf {I}}-\frac{1}{m} {\textbf {11}}') \end{aligned}$$

where ${\textbf {D}}=(d_{ij})$ is an $m\times m$ taxon-based distance or dissimilarity matrix (e.g., weighted, or unweighted UniFrac distance or the Bray-Curtis dissimilarity) [19, 20], ${\textbf {D}}^{(2)}$ is the element-wise squared distance matrix, **I** is the $m\times m$ identity matrix, **1** in $\frac{1}{m} {\textbf {11}}'$ is the $m\times 1$ vector of ones.

### Variance structure for sample-related random effect

The random effect **u** captures the combined small effects of all markers, with the assumption that comparable samples are believed to capture similar effects. Consequently, we have the opportunity to integrate sample similarity into the modeling of variance-covariance matrix of **u**. This matrix of pairwise similarities between individuals is defined as the kernel matrix **K** [21]. For microbiome composition data, the OTUs are related by a phylogenetic tree. Kernels that exploit the degree of divergence between different sequences can be much more powerful than similarity measures that ignore the phylogenetic-tree information [21]. To measure similarities between the microbiome compositions among subjects, we construct the kernel matrix **K** through the following transformation of sample distance metrics [22]:

9 $$\begin{aligned} {\textbf {K}}=-\frac{1}{2}({\textbf {I}}-\frac{1}{n} {\textbf {11}}'){\textbf {D}}^{(2)}({\textbf {I}}-\frac{1}{n} {\textbf {11}}') \end{aligned}$$

where ${\textbf {D}}=(d_{ij})$ is an $n\times n$ sample-based pairwise distance matrix (UniFrac distance or the Bray-Curtis dissimilarity). Here, **I** is the $n\times n$ identity matrix, **1** in $\frac{1}{n} {\textbf {11}}'$ is the $n\times 1$ vector of ones.

Note that we use ${\textbf {D}}^{(2)}$ to calculate the weight matrix **W** and the kernel matrix **K**. ${\textbf {D}}^{(2)}$ differ in these two formulas: one represents an $m\times m$ matrix of squared Euclidean distances between taxa, while the other represents an $n\times n$ matrix of squared Euclidean distances between samples.

### Algorithm

The proposed method can be implemented in **Stan** using brms package. The brms package provides an interface for defining and fitting Bayesian models, making use of the powerful Stan platform, which is a C++ package for obtaining full Bayesian inference [23]. The package incorporates a highly efficient Markov chain Monte Carlo (MCMC) algorithm, specifically the Hamiltonian Monte Carlo (HMC) method and its adaptive version known as the No-U-Turn sampler (NUTS) [11]. The Hamiltonian Monte Carlo algorithm generates posterior samples for all the parameters from the joint posterior distribution, which is defined by the likelihood function and the prior distributions, i.e.,

10 $$\begin{aligned} p(\beta _0,\beta ^*,\tau ,\lambda ,c^2,u|y,z)\propto p(y|\eta ,\phi )p(\sum _{j=1}^m\beta ^*)p(\beta _0)p(\beta ^*)p(\tau )p(\lambda )p(c^2)p(u) \end{aligned}$$

The posterior sample is then used to summarize the posterior distribution for each parameter in various ways. In our simulation studies and real data analyses, we used the posterior mean as point estimate.

### Evaluation of predictive performance

There are various measures to assess the performance of the fitted model, including deviance, a generic way of measuring the model’s quality defined as $-2\sum _{i=1}^{n}log(y_i|\eta _i,\phi )$, mean squared error (MSE), defined as $\frac{1}{n}\sum _{i=1}^{n}(y_i-\hat{y}_i)^2$. For continuous response, R squared and mean absolute error (MAE), defined as $\frac{1}{n}\sum _{i=1}^{n}|y_i-\hat{y}_i|$ are considered. For binary response, two additional measures can be employed: area under the ROC curve (AUC) and misclassification rate, which is defined as $\frac{1}{n}\sum _{i=1}^{n}I(|y_i-\hat{y}_i|>0.5)$ where $I(|y_i-\hat{y}_i|>0.5)=1$ if $|y_i-\hat{y}_i|>0.5$ and $I(|y_i-\hat{y}_i|>0.5)=0$ if $|y_i-\hat{y}_i|\le 0.5$ [24].

To evaluate the predictive performance of the proposed model, a general way is to fit the model using a data set (training data), and then calculate the above measures with an external data set (validation data). If external data is not available, the commonly used method is cross-validation. Vehtari et al. developed an approximate leave-one-out cross-validation method, which uses the posterior samples from the fitted model to calculate the cross-validated quantities without the need to refit the model [9, 25].

## Simulation studies

### Simulation design

We use simulation to test our proposed approach BCGLMM. The proposed method was tested for both continuous and binary outcomes.

We first generated a $400 \times m \,(m=100,300,500)$ data matrix ${\textbf {U}}=(u_{ij})$ from a multivariate normal distribution $N_m(\theta ,\Sigma )$, and then used the transformation $x_{ij}=e^{(u_{ij} )}/\sum _{k=1}^{m}e^{(u_{ik})}$ to obtain the relative abundance matrix ${\textbf {X}}=(x_{ij})$. Two groups of effects $\beta _j^*$, a small number of moderate effects and a larger number of small effects, are considered. For the small number of moderate effects, we considered three sets: one with 0 effects, one with 6 effects and the other with 12 effects. In the first set, there are no moderate effects. In the second set, the true coefficients are $\beta _j^*$, those $j=16+2\iota ,\iota =1,2,\cdots ,6$. The corresponding 6 nonzero coefficients are $\beta _j^* = (2.08, 1.50, -1.16, -0.86, -2.12,0.56)$. In the third set, the true variables are $\beta _j^*$, where $j=16+2\iota ,\iota =1,2,\cdots ,12$. The corresponding 12 nonzero coefficients are $\beta _j^* = (2.08, -1.41, -1.39, -1.15, 2.12, 0.51, 1.31, -0.95, -0.86, 1.93, -1.34, -0.85)$. For the larger number of small effects, we explored different quantities of small effects, with their proportion set at three levels corresponding to the total number of predictors: 0.2, 0.5, and 0.7. These small effects were generated from a Normal distribution with a mean of 0 and a standard deviation of 0.2. The sum of the effects in the two groups equals 0. In total, we considered 27 scenarios for both continuous and binary outcomes. For each scenario, we replicated the simulation 100 times and summarized the results across these replicates.

We let $\theta _j=log(0.5m)$ among the moderate effects and 0 otherwise. Among the moderate effect predictors, the covariance is assumed to be $\Sigma _{ij}=0.75-0.015|i-j|$, while among the small effect predictors, the covariance is assumed to be $\Sigma _{ij}=0.25-0.00015|i-j|$ [8]. This means the correlation between two covariates is negatively proportional to their distance. Next, we generated the normal continuous outcome from the univariate normal distribution $N(\eta _i,1.6^2)$, where $\eta _i=\sum _{j=1}^{m}log(x_{ij})\beta _j^*$. For the binary response, we dichotomized these continuous responses at median by setting individuals with 50% largest continuous response as case ($y_i=1$) and the other individuals as control ($y_i=0$) [26].

In this study, we applied our proposed method in three distinct ways: BCGLMM, which considers both the sample-related random effect and predictor relatedness simultaneously; BCGLM, which focuses solely on the predictor correlations [9]; and BGLM, which ignores both the random effect and predictor correlations. This allowed us to assess how predictor interrelationships and random effects influence model fitting outcomes. As we know that for now, there is no such paper that has addressed the compositional challenges and random effects, so we did not consider other methods.

Since our application focus on the relationship between **y** and **X**, not on interpreting estimates of $\beta _j^*$, we will assess the prediction performance of simulation. Deviance, R squared, mean absolute error (MAE), and mean squared error (MSE) will be reported for continuous responses; deviance, area under the ROC curve (AUC), MSE, and misclassification rate (MR) will be reported for binary responses. Note that ideal models will have lower values for deviance, MSE, MAE, misclassification rate, and higher values for AUC and R squared.

Bray-Curtis distance was employed to calculate dissimilarity matrix ${\textbf {D}}$ between taxa/ sample in our simulation [19]. To evaluate the accuracy of our prognostic model, we utilized the leave-one-out cross-validation technique to assess its prediction performance. We implemented the approximate Bayesian leave-one-out cross-validation method [25], which is computationally more efficient than exact leave-one-out cross-validation since it only requires one evaluation of the model rather than refitting the model *n* times.

All statistical analyses were performed using **R** software (version 4.0.5). Our proposed method, BCGLMM, **R** function BCGLM and BGLM were implemented with the brms package (version 2.17.0).

### Simulation result

Tables 1 and 2 present the prediction performance for continuous and binary outcomes, respectively, in scenarios involving 6 moderate effects. Table 1 demonstrates that BCGLM, which accounts for predictor intercorrelation, outperforms BGLM across all scenarios with lower deviance and lower mean squared error (MSE). This emphasizes the significance of considering interrelationships among predictors. In scenarios where $m=100$, compared to BCGLM and BGLM, BCGLMM that incorporates sample-related random effect did not exhibit a clear advantage when the proportion $\in (0.2, 0.5)$. However, when the proportion is set to 0.7, BCGLMM begins to show better performance. As $m \in (300, 500)$, BCGLMM consistently outperforms the other two methods, and this trend becomes more pronounced as the proportion of small effects increases. Specifically, when $m=500$, BCGLMM displays significantly higher R-squared values and much lower deviance compared to the other methods. For instance, with $m=300$ and a proportion of small effects set at 0.7, the model using BCGLMM has a deviance of 2388.5 and an R-squared value of 0.814, in contrast to 2621.4 and 0.787 for BGLM. The result aligns with expectations, as the random effect **u** in BCGLMM captures the combined small effects of all markers, and these effects become more evident as the proportion of small effects increases.

**Table 1:** Model performance comparison between the proposed methods for continuous outcomes with 6 moderate effects

**m**	Proportion$\ddagger$	Model	Deviance	$R^2$	MSE	MAE
100	0.2	BGLM	1907.1	0.670	2.930	1.363
		BCGLM	1902.9	0.671	2.919	1.360
		BCGLMM	1911.2	0.669	2.940	1.365
	0.5	BGLM	1976.7	0.714	3.104	1.402
		BCGLM	1971.7	0.715	3.091	1.399
		BCGLMM	1972.7	0.715	3.094	1.401
	0.7	BGLM	2016.5	0.740	3.203	1.424
		BCGLM	2012.1	0.741	3.192	1.421
		BCGLMM	2009.9	0.742	3.186	1.422
300	0.2	BGLM	2147.6	0.701	3.531	1.503
		BCGLM	2130.7	0.704	3.488	1.494
		BCGLMM	2089.8	0.712	3.386	1.478
	0.5	BGLM	2450.3	0.751	4.287	1.660
		BCGLM	2430.6	0.754	4.238	1.651
		BCGLMM	2286.1	0.775	3.877	1.584
	0.7	BGLM	2621.4	0.787	4.715	1.749
		BCGLM	2596.8	0.790	4.654	1.737
		BCGLMM	2388.5	0.814	4.133	1.637
500	0.2	BGLM	2282.8	0.720	3.869	1.583
		BCGLM	2244.7	0.728	3.773	1.564
		BCGLMM	2103.6	0.752	3.421	1.488
	0.5	BGLM	2839.0	0.778	5.260	1.849
		BCGLM	2765.4	0.786	5.075	1.816
		BCGLMM	2300.6	0.832	3.913	1.576
	0.7	BGLM	3213.1	0.788	6.195	2.009
		BCGLM	3096.6	0.798	5.903	1.961
		BCGLMM	2521.0	0.846	4.464	1.676

$\ddagger$ Proportion: the proportion of small effects corresponding to the total predictors. BCGLMM, which considers both the sample-related random effect and predictor correlations simultaneously; BCGLM, which focuses solely on the predictor correlations; and BGLM, which ignores both the random effect and predictor correlations

A similar trend was observed for binary outcomes, as presented in Table 2. BCGLM, which incorporated predictor correlations, outperformed BGLM, exhibiting lower deviance and higher AUC. This trend became more apparent as the number of predictors and the proportion of small effects increased. In contrast to continuous outcomes, when $m=300$, BCGLMM demonstrated superior performance compared to BCGLM and BGLM for binary outcomes.

**Table 2:** Model performance comparison between the proposed methods for binary outcomes with 6 moderate effects

**m**	Proportion$\ddagger$	Model	Deviance	AUC	MSE	MR
100	0.2	BGLM	357.3	0.869	0.146	0.217
		BCGLM	354.0	0.872	0.145	0.213
		BCGLMM	361.3	0.866	0.148	0.219
	0.5	BGLM	335.9	0.884	0.137	0.200
		BCGLM	333.4	0.886	0.136	0.198
		BCGLMM	335.9	0.884	0.136	0.200
	0.7	BGLM	333.9	0.885	0.136	0.200
		BCGLM	330.9	0.888	0.134	0.197
		BCGLMM	328.7	0.889	0.133	0.196
300	0.2	BGLM	360.6	0.864	0.148	0.220
		BCGLM	353.2	0.871	0.144	0.215
		BCGLMM	350.0	0.874	0.142	0.208
	0.5	BGLM	355.3	0.867	0.146	0.215
		BCGLM	348.6	0.872	0.142	0.209
		BCGLMM	333.3	0.887	0.135	0.196
	0.7	BGLM	336.0	0.881	0.137	0.200
		BCGLM	327.7	0.888	0.133	0.193
		BCGLMM	312.1	0.903	0.124	0.176
500	0.2	BGLM	360.4	0.866	0.147	0.218
		BCGLM	344.3	0.880	0.140	0.203
		BCGLMM	350.3	0.876	0.142	0.206
	0.5	BGLM	344.8	0.878	0.140	0.203
		BCGLM	328.6	0.893	0.132	0.187
		BCGLMM	332.3	0.891	0.133	0.190
	0.7	BGLM	346.3	0.875	0.140	0.203
		BCGLM	321.7	0.898	0127	0.177
		BCGLMM	327.3	0.895	0.131	0.184

$\ddagger$ Proportion: the proportion of small effects corresponding to the total predictors. MR: misclassification rate. BCGLMM, which considers both the sample-related random effect and predictor correlations simultaneously; BCGLM, which focuses solely on the predictor correlations; and BGLM, which ignores both the random effect and predictor correlations

**Fig. 1:**
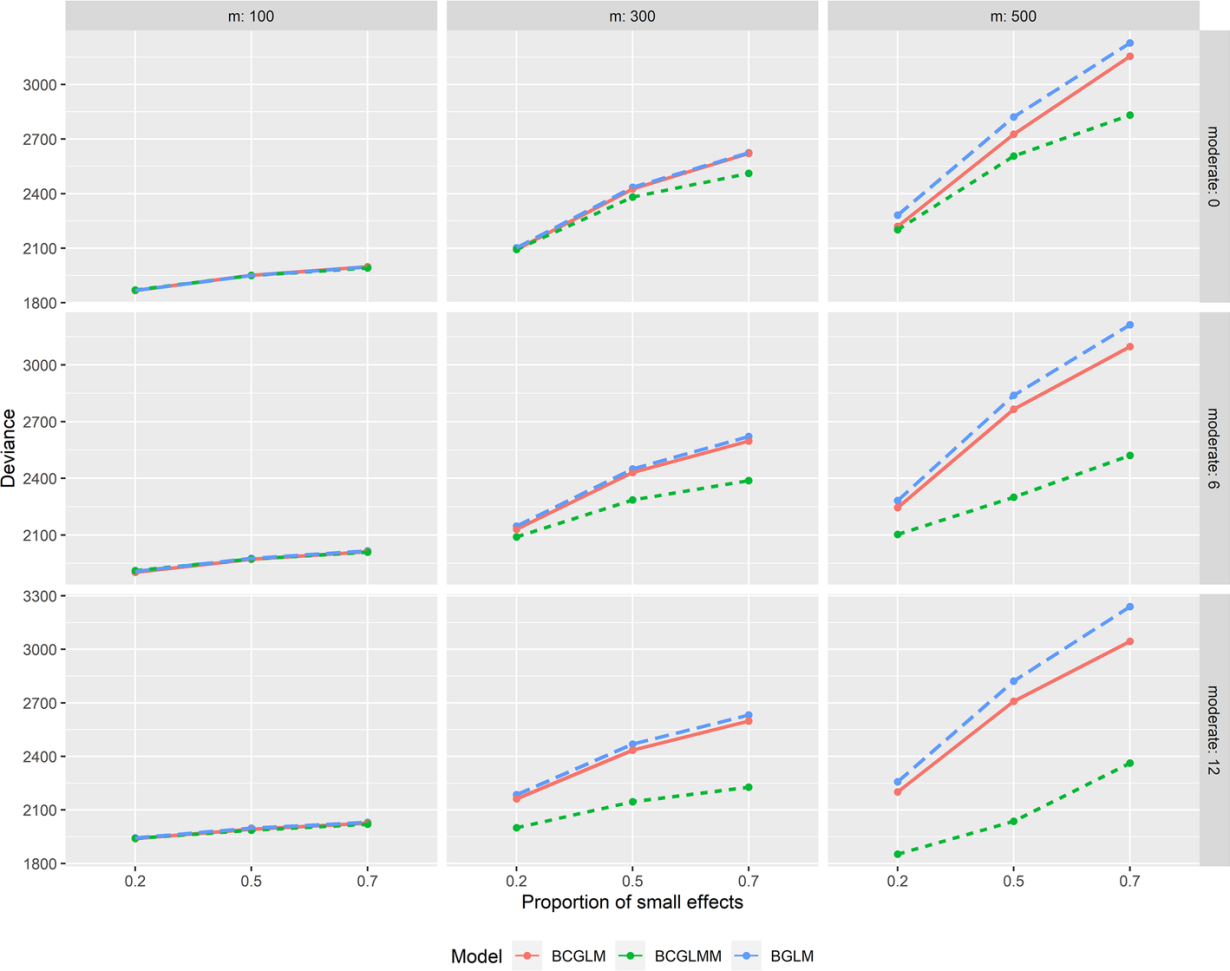
Deviance comparison among the three methods in various scenarios for continuous outcomes. BCGLMM considers both the sample-related random effect and predictor correlations simultaneously, BCGLM focuses solely on predictor correlations, and BGLM ignores both the random effect and predictor correlations

**Fig. 2:**
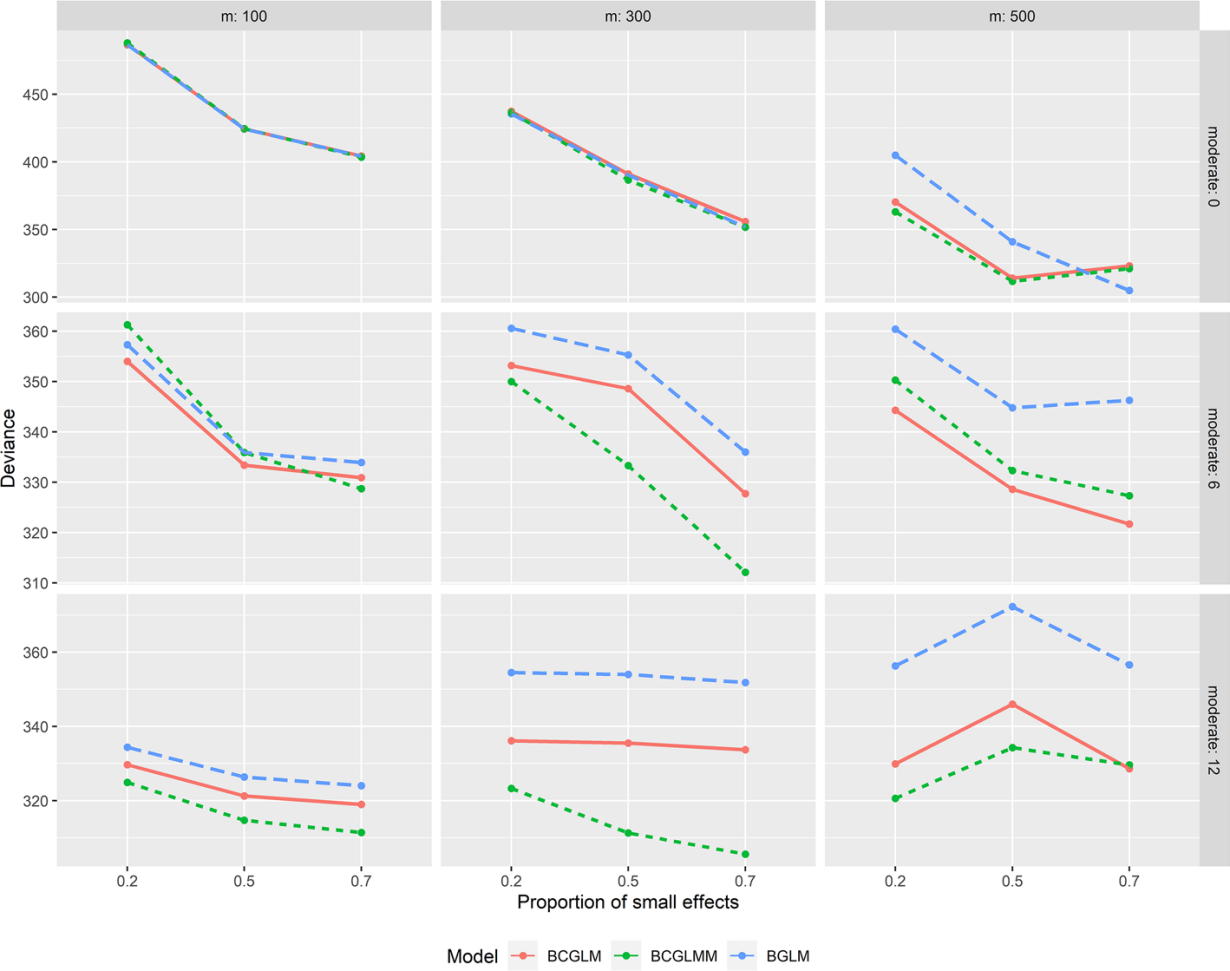
Deviance comparison among the three methods in various scenarios for binary outcomes. BCGLMM considers both the sample-related random effect and predictor correlations simultaneously, BCGLM focuses solely on predictor correlations, and BGLM ignores both the random effect and predictor correlations

In Supporting information Tables 1 and 2, we present the prediction performance for continuous and binary outcomes, respectively, in set 1 when there are no moderate effects. Similarly, in Table 3 and Table 4, we display the prediction performance for continuous and binary outcomes, respectively, in scenarios involving 12 moderate effects. A very similar trend to that observed in scenarios with 6 moderate effects is seen for continuous outcomes. As depicted in Fig. 1, the superiority of BCGLMM becomes even more pronounced for continuous outcomes in scenarios with 12 moderate effects, particularly for $m \in (300, 500)$. In the case of binary outcomes, BCGLMM consistently outperforms the other two methods in all the scenarios with 12 moderate effects. Figure 2, which compares the deviance for binary outcomes, further emphasizes the increased superiority of BCGLMM in scenarios with 12 moderate effects compared to scenarios with 6 moderate effects.

In summary, the methods exhibit good convergence when $m \in (100, 300)$ for both continuous and binary responses. When $m = 500$, a modification is introduced by replacing the half-Cauchy priors for the local parameters $\lambda _j$ with half-*t* priors with 3 degrees of freedom. This adjustment effectively addresses the occurrence of divergent transitions in the No-U-Turn Sampler (NUTS) due to the heavy-tailed nature of the Cauchy priors. For binary responses, we also replace the global parameters $\tau$ with half-*t* priors with 3 degrees of freedom to avoid problems arising from data separation in logistic regression [14].

## Real data application

We showcased the efficacy of our proposed method by implementing it on a publicly available American Gut Project questionnaire. The project, established to advance our knowledge of human microbiomes, involves participants who provided fecal, oral, and/or integumentary body samples. Additionally, participants completed a self-administered questionnaire covering demographics, lifestyle preferences, medical history, and dietary patterns. The project offers open-source, open-access 16 S rRNA data categorized by rarefaction depth and sequence trim length [27].

Our study includes 1002 species for 4684 samples taken from the fecal body site. The self-administered questionnaire contains 204 host characteristics, including the diagnoses of inflammatory bowel disease (IBD), with 165 of them are IBD cases defined by responses “Diagnosed by a medical professional (doctor, physician assistant)” and 4519 IBD control defined by “I do not have this ailment”.

Our analysis aimed to predict IBD event using the 1002 species as predictors. Zero count was replaced with 0.5, which is commonly used in microbiome data analysis [8], before being divided by the sum to obtain the species composition. The proposed method, BCGLMM (mixed effects logistic regression with samplewise random effect and structured regularized horseshoe prior here), was applied based on the log-transformed compositions. For comparison, we also fitted the BCGLM (logistic regression with structured regularized horseshoe prior) and BGLM (logistic regression with regularized horseshoe prior) models.

To evaluate the accuracy of our prognostic model, we utilized the approximate Bayesian leave-one-out cross-validation method [25]. Model performance was evaluated using the area under the ROC curve (AUC). The cross-validated AUC values for BCGLMM, BCGLM and BGLM were 0.702, 0.687 and 0.672, respectively. These results demonstrate the effectiveness of these models in predicting IBD. Notably, our BCGLMM approach, which considers both sample-related random effects and predictor correlations simultaneously, outperformed the other methods, underscoring the strong predictive power of our model and its potential to enhance the diagnosis and treatment of inflammatory bowel disease.

## Discussion

We have introduced a novel statistical and computational method, Bayesian Compositional Generalized Linear Mixed Models for Analyzing Microbiome Data (BCGLMM). This method not only addresses the compositional constraint in estimating the regression coefficients but also incorporates phylogenetic relationships among bacterial taxa. We operate under the biologically plausible assumption that closely related taxa share similar effects on the clinical trait.

BCGLMM presents a novel approach to modeling microbiome data, enabling the incorporation of both a small number of individually substantial genetic effects and the collective impact of numerous small genetic factors. The balance between these two types of effects is deduced from the available data. This study marks the first attempt to integrate sparse models and mixed models for the analysis of compositional microbiome data. Prior research has predominantly concentrated on sparse settings, assuming that only a few predictors among the high-dimensional set are nonzero. In our work, we introduce a sparsity-inducing prior, the regularized horseshoe prior, which effectively selects for moderate effects. By incorporating a mixed model, the random effect term efficiently captures sample-related small effects by considering sample similarities as a variance-covariance matrix. This innovative approach aims to provide a more comprehensive understanding of microbiome data, enhancing our ability to identify relevant factors and relationships.

The flexibility and versatility of BCGLMM extend beyond the analysis of continuous outcomes, making it a valuable tool for various types of data. In addition to its successful application to binary outcomes, as demonstrated in both simulation and real data analyses, BCGLMM can be readily extended to accommodate other types of outcomes, such as ordinal, count, or survival data. This adaptability underscores the broad utility of our innovative approach, ensuring its relevance in a wide range of research areas and applications.

Our work builds upon prior studies of our group [9, 15], further expanding the capabilities of this modeling framework. While BCGLMM offers a robust solution for analyzing microbiome data, it is essential to acknowledge that, like many posterior sampling-based methods, it carries a substantial computational burden. This burden stems from the demands on memory and CPU time, which can be significant in large-scale studies. Despite these challenges, BCGLMM represents a significant advancement in the field of microbiome research, providing a powerful and flexible tool for addressing complex data structures with the ability to renew valuable insights.

## Conclusion

We have introduced a novel statistical and computational method for disease prediction that utilizes compositional microbiome data. This method effectively addresses several critical challenges inherent in compositional microbiome data, including its compositional structure, high dimensionality, and phylogenetic relationships. By employing a mixed model approach, our method enhances disease prediction by effectively combining small effects, thereby improving predictive accuracy. This represents the first time that these specific challenges have been systematically addressed in the context of disease prediction using microbiome data.

## Supplementary Information

Supplementary Material 1.

## Data Availability

To support translational science, we have released our proposed method as an R package, accessible at https://github.com/Li-Zhang28/BCGLMM. This inclusive package encompasses simulation code, data analysis, and real dataset examples, serving as a valuable resource for researchers keen on applying our approach.
