# Supplementary material for: Bayesian compositional generalized linear mixed models for disease prediction using microbiome data
Source: BMC Bioinformatics. 2025 Apr 5;26:98. doi: 10.1186/s12859-025-06114-3 (PMC11971746; doi:10.1186/s12859-025-06114-3)
Supplement: Supplementary file 1 — Supplementary Material 1. [file 12859_2025_6114_MOESM1_ESM.pdf]

Supporting Information for "Bayesian Compositional Generalized Linear Mixed Models for Disease Prediction using Microbiome Data"

Table 1: Model performance comparison between the proposed methods for continuous outcomes with 0 moderate effects

	proportion		Deviance	R2	MSE	MAE
m=100						
	0.2	BGLM	1867.6	0.279	2.831	1.340
		BCGLM	1867.9	0.279	2.831	1.340
		BCGLMM	1869.8	0.277	2.836	1.341
	0.5	BGLM	1950.3	0.482	3.038	1.387
		BCGLM	1951.3	0.481	3.040	1.388
		BCGLMM	1949.8	0.482	3.036	1.387
	0.7	BGLM	1996.7	0.549	3.154	1.412
		BCGLM	1998.1	0.549	3.157	1.413
		BCGLMM	1990.9	0.552	3.139	1.409
m=300						
	0.2	BGLM	2102.6	0.477	3.418	1.478
		BCGLM	2093.2	0.480	3.395	1.473
		BCGLMM	2093.3	0.480	3.395	1.473
	0.5	BGLM	2435.4	0.641	4.250	1.654
		BCGLM	2425.4	0.643	4.225	1.650
		BCGLMM	2380.8	0.652	4.114	1.629
	0.7	BGLM	2624.3	0.714	4.722	1.743
		BCGLM	2621.8	0.715	4.716	1.742
		BCGLMM	2511.3	0.731	4.440	1.692
m=500						
	0.2	BGLM	2282.1	0.558	3.867	1.579
		BCGLM	2219.7	0.575	3.711	1.550
		BCGLMM	2202.0	0.580	3.667	1.542
	0.5	BGLM	2821.9	0.720	5.217	1.842
		BCGLM	2726.3	0.733	4.977	1.798
		BCGLMM	2606.4	0.750	4.678	1.743
	0.7	BGLM	3229.1	0.737	6.234	2.016
		BCGLM	3155.3	0.745	6.050	1.985
		BCGLMM	2832.0	0.779	5.242	1.838

proportion: the proportion of small effects corresponding to the total predictors. BCGLMM, which considers both the sample-related random effect and predictor correlations simultaneously; BCGLM, which focuses solely on the predictor correlations; and BGLM, which ignores both the random effect and predictor correlations.

Table 2: Model performance comparison between the proposed methods for binary outcomes with 0 moderate effects

	proportion		Deviance	AUC	MSE	MR
m=100						
	0.2	BGLM	486.6	0.713	0.211	0.340
		BCGLM	486.7	0.713	0.211	0.341
		BCGLMM	488.0	0.710	0.212	0.343
	0.5	BGLM	424.3	0.797	0.179	0.276
		BCGLM	424.5	0.797	0.179	0.276
		BCGLMM	424.6	0.796	0.179	0.276
	0.7	BGLM	404.2	0.819	0.169	0.256
		BCGLM	404.3	0.819	0.169	0.256
		BCGLMM	403.5	0.820	0.169	0.255
m=300						
	0.2	BGLM	435.6	0.783	0.185	0.285
		BCGLM	437.3	0.780	0.186	0.286
		BCGLMM	436.4	0.780	0.186	0.285
	0.5	BGLM	390.3	0.832	0.162	0.243
		BCGLM	391.1	0.831	0.163	0.243
		BCGLMM	386.6	0.836	0.160	0.239
	0.7	BGLM	351.9	0.865	0.144	0.212
		BCGLM	355.9	0.862	0.146	0.216
		BCGLMM	351.8	0.867	0.144	0.212
m=500						
	0.2	BGLM	405.0	0.815	0.169	0.256
		BCGLM	370.3	0.846	0.151	0.222
		BCGLMM	363.1	0.852	0.148	0.217
	0.5	BGLM	341.0	0.865	0.128	0.175
		BCGLM	314.0	0.904	0.124	0.170
		BCGLMM	311.8	0.906	0.123	0.167
	0.7	BGLM	305.0	0.865	0.141	0.161
		BCGLM	323.1	0.892	0.129	0.180
		BCGLMM	321.1	0.894	0.128	0.178

proportion: the proportion of small effects corresponding to the total predictors. MR: misclassification rate

BCGLMM, which considers both the sample-related random effect and predictor correlations simultaneously; BCGLM, which focuses solely on the predictor correlations; and BGLM, which ignores both the random effect and predictor correlations.

Table 3: Model performance comparison between the proposed methods for continuous outcomes with 12 moderate effects

	proportion		Deviance	R2	MSE	MAE
m=100						
	0.2	BGLM	1943.7	0.729	3.021	1.385
		BCGLM	1940.0	0.730	3.012	1.383
		BCGLMM	1940.7	0.730	3.014	1.384
	0.5	BGLM	1997.7	0.762	3.156	1.413
		BCGLM	1992.6	0.763	3.143	1.411
		BCGLMM	1985.8	0.765	3.126	1.408
	0.7	BGLM	2030.9	0.779	3.239	1.434
		BCGLM	2026.3	0.780	3.227	1.431
		BCGLMM	2020.0	0.781	3.212	1.429
m=300						
	0.2	BGLM	2185.3	0.743	3.625	1.527
		BCGLM	2162.7	0.747	3.568	1.517
		BCGLMM	2000.4	0.775	3.163	1.431
	0.5	BGLM	2468.9	0.779	4.334	1.672
		BCGLM	2435.4	0.783	4.250	1.657
		BCGLMM	2146.0	0.820	3.527	1.506
	0.7	BGLM	2632.2	0.808	4.742	1.753
		BCGLM	2598.6	0.811	4.658	1.738
		BCGLMM	2227.4	0.849	3.730	1.552
m=500						
	0.2	BGLM	2258.2	0.768	3.807	1.571
		BCGLM	2200.9	0.777	3.664	1.543
		BCGLMM	1852.5	0.829	2.793	1.332
	0.5	BGLM	2822.1	0.795	5.217	1.851
		BCGLM	2709.0	0.807	4.934	1.799
		BCGLMM	2036.9	0.868	3.254	1.413
	0.7	BGLM	3240.1	0.799	6.262	2.019
		BCGLM	3045.4	0.815	5.775	1.943
		BCGLMM	2363.2	0.869	4.070	1.580

proportion: the proportion of small effects corresponding to the total predictors. BCGLMM, which considers both the sample-related random effect and predictor correlations simultaneously; BCGLM, which focuses solely on the predictor correlations; and BGLM, which ignores both the random effect and predictor correlations.

Table 4: Model performance comparison between the proposed methods for binary outcomes with 12 moderate effects

	proportion		Deviance	AUC	MSE	MR
m=100						
	0.2	BGLM	334.4	0.887	0.135	0.199
		BCGLM	329.7	0.890	0.133	0.196
		BCGLMM	324.9	0.895	0.131	0.187
	0.5	BGLM	326.4	0.892	0.132	0.195
		BCGLM	321.3	0.895	0.130	0.192
		BCGLMM	314.7	0.902	0.126	0.183
	0.7	BGLM	324.0	0.893	0.131	0.191
		BCGLM	319.0	0.897	0.129	0.186
		BCGLMM	311.4	0.903	0.125	0.179
m=300						
	0.2	BGLM	354.5	0.870	0.145	0.212
		BCGLM	336.1	0.885	0.136	0.198
		BCGLMM	323.3	0.898	0.129	0.185
	0.5	BGLM	354.0	0.869	0.145	0.213
		BCGLM	335.5	0.885	0.136	0.198
		BCGLMM	311.3	0.908	0.123	0.174
	0.7	BGLM	351.8	0.871	0.143	0.209
		BCGLM	333.7	0.886	0.135	0.193
		BCGLMM	305.6	0.913	0.120	0.166
m=500						
	0.2	BGLM	356.3	0.871	0.145	0.211
		BCGLM	329.9	0.896	0.131	0.184
		BCGLMM	320.6	0.902	0.127	0.178
	0.5	BGLM	372.3	0.856	0.153	0.225
		BCGLM	346.0	0.882	0.139	0.915
		BCGLMM	334.3	0.893	0.134	0.188
	0.7	BGLM	356.6	0.867	0.145	0.208
		BCGLM	328.6	0.890	0.130	0.182
		BCGLMM	329.6	0.896	0.131	0.183

proportion: the proportion of small effects corresponding to the total predictors. MR: misclassification rate

BCGLMM, which considers both the sample-related random effect and predictor correlations simultaneously; BCGLM, which focuses solely on the predictor correlations; and BGLM, which ignores both the random effect and predictor correlations.
